# Sexual dysfunction in women with genital warts: a systematic review

**DOI:** 10.1186/s12905-022-02073-6

**Published:** 2022-12-12

**Authors:** Mohadese Adeli, Lida Moghaddam-Banaem, Shadab Shahali

**Affiliations:** 1 Department of Reproductive Health and Midwifery, Faculty of Medical Sciences, Tarbiat Modares University, Al-E-Ahmad Highway, Tehran, Iran; 2 Department of Midwifery, School of Nursing and Midwifery, Torbat Heydariyeh University of Medical Sciences, Torbat Heydariyeh, Iran

**Keywords:** Sexual dysfunction, Genital wart, HPV, Condyloma acuminate, Psychosexual impact

## Abstract

**Purpose:**

To provide an understanding of the changes in sexual function in women with genital warts.

**Methods:**

In this review study, databases searched included: PubMed, Science Direct, Scopus, Web of Science, Cochrane Library of Systematic Reviews, Google Scholar, ProQuest, Wiley, and Highwire Press. No study design limitations were applied to the initial search, and qualitative and quantitative studies published between 2005 and 2021 were included.

**Results:**

19 articles were selected and analyzed narratively. The quality of the studies was almost good. The findings were classified into three groups: The prevalence of sexual dysfunction in women with genital warts (GWs) and Human Papillomavirus (HPV); Types of sexual dysfunction in women with genital warts, and associated factors; Psychosexual effects of genital warts.

**Conclusion:**

This study provides up-to-date evidence of the changes in sexual function in women with genital warts. Although the results of most studies showed that women with genital warts experienced sexual dysfunction in almost all dimensions, differences in study design and study population made it difficult to determine the specific type of disorder such as libido, or arousal disorders in these women. Based on the findings of this review, more research in this field is recommended for the future.

*Systematic review registration*: (PROSPERO: CRD42020188584, https://www.crd.york.ac.uk/prospero/display_record.php?RecordID=188584).

**Supplementary Information:**

The online version contains supplementary material available at 10.1186/s12905-022-02073-6.

## Background

Genital warts (GWs) are common complications of human papillomavirus (HPV) [1, 2]. In a review study performed by Patel et al. in 2013, the annual prevalence of GWs in the world was generally reported in men and women as160–289 per 100,000 [3, 4]. The ages at peak incidence of GWs in men and women are 25–29, and 20–24 years old respectively [5]. In Iran, accurate information on the prevalence of GWs is not available, but GWs diagnosis has been reported among people aged 20–30 years old [3].

The most common factors affecting the incidence of GWs include: age, sex, level of education, race, marital status, age at first sexual contact, new sexual partner, number of sexual partners, condom use, smoking, family history, socioeconomic status, other sexually transmitted infections, oral contraceptive pills, and alcohol use; some of which have been confirmed and some are still under debate [3, 6–8].

According to some studies, HPV and GWs have many physical and psychological effects on patients. Initial reactions of patients include anger, depression, isolation, shame, and guilt [9]. GWs may affect sexual life, self-image, self-esteem, emotions, daily activities, and the quality of life, because of pain and discomfort, anxiety, and depression [10–12]. Almost all studies confirm that GWs threaten people's sexual health and cause obvious changes in their sex lives.

Female sexual dysfunctions (FSD) include dyspareunia, female orgasmic disorder, female sexual arousal disorder, vaginismus, and sexual difficulties from underlying medical causes. The estimates of female sexual dysfunctions' prevalence vary greatly in different studies, but the American Psychological Association has suggested that more than 40% of women in the United States experience FSD [13]. Prevalence of sexual dysfunction is higher in women than men [14].

Sexually transmitted infections (STIs) can threaten a couple's sexual function. GWs directly affect women's sexual function, as they not only cause pain and discomfort for the patients, but also reduce their sexual desire [15, 16]. Concerns about the spread of lesions or recurrence of lesions, fear of transmitting the disease to the spouse, worry about the spouse's infidelity, lack of sufficient sexual knowledge and skills, mental conflict, confusion and restlessness after diagnosis, are among the reasons for the decrease in sexual desire, arousal, and orgasm in women with GWs. Sometimes these concerns are passed on to the spouses, and indirectly affect their sexual arousal and orgasm too [17].

Lack of sexual satisfaction causes physical and mental stress. A study in Australia reported that 88% of men and 80% of women believe that healthy sex is very effective in improving the sense of vitality [18].

There are few studies about sexual dysfunction in women with GWs. Although some studies showed that GWs could cause sexual dysfunction in patients [16, 17, 19–21], others reported that genital warts did not have negative effects on sexual function, or stated that only some dimensions of sexual function were affected [22].

Considering the increasing prevalence of GWs among women and their dire consequences in a couple's sexual function, and the inadequate number of analytical studies, or systematic reviews of related studies, this review was performed to answer the following questions: What types of sexual dysfunction occur in women with genital warts, and what are their frequencies? What are the physical, emotional, psychological, and social effects of genital warts on sexual function of women with genital warts?

### Objective

This review aims to provide an understanding of sexual function in women with genital warts.

## Methods

This is a systematic review with narrative analysis as there was a combination of quantitative and qualitative studies assessed. All quantitative studies were cross-sectional with no clinical trial study, and there was a clear heterogeneity in the studies’ methodology, so a meta-analysis could not be performed.

This review study was registered with Prospero (PROSPERO: CRD42020188584, https://www.crd.york.ac.uk/prospero/display_record.php?RecordID=188584), and PRISMA guidelines were followed (Additional file 1; Prisma checklist).

### Eligibility criteria

No study design limitations were applied to the initial search, and both qualitative and quantitative studies published between 2005 and 2021 were included. Studies were included if they mentioned search terms mentioned below. Reviews, narrative reviews, abstracts, comments, and editorial articles were excluded. Articles were also excluded if they were not in English or Persian, if they studied cervical cancer or cervical intraepithelial neoplasia, or if they were about sexual dysfunction in men, Trans genders, and children.

### Information sources

The databases searched included: PubMed, Science Direct, Scopus, Web of Science, Cochrane Library of Systematic Reviews, Google Scholar, ProQuest, Wiley, and Highwire Press. Also, additional articles were identified by searching for grey literatures using OpenGrey (www.opengrey.eu).

### Search strategy

Search terms included: (a) genital warts (b) HPV (c) condyloma acuminata (d) sexual dysfunction (sexual desire, arousal, lubrication, orgasm, satisfaction, and pain during intercourse) and (e) psychosexual impacts. To increase the search abilities, Boolean strategy were incorporated (Additional file 2: Search Strategy).

### Study records and selection process

Based on the keywords and inclusion criteria, relevant articles were selected from each database. After removing duplicates, a folder containing the obtained articles was created, and shared among the researchers to read and check.

Titles were screened by a reviewer (MA). Two reviewers (MA and SS) screened abstracts of the included papers (agreement rate = 90%). Screening was based on a Data Extraction Form (Additional file 3) designed by the authors. If an article could not be evaluated based on the abstract, the full text of the article was reviewed. If there was a disagreement among the reviewers, it was resolved by discussion among the researchers, and if not resolved, the third researcher (LM) got the consensus.

#### Quality assessment and risk of bias

A modified version of the National Institute for Health and Care Excellence (NICE (was used to evaluate the quality of quantitative and qualitative studies. the quality of the studies is summarized in Tables 1 and 2.

**Table 1:** Characteristics of quantitative studies measuring sexual dysfunction and psychosexual outcomes included in the review

References	Country	Age (y)	Psychosexual outcomes measured	Number of participants	Survey instrument	Time of data collection	Study population	Comparison groups	Quality assessment score internal validity/external
El-esawy et al. [20]	Egypt	18–45	(Sexual desire, arousal, lubrication, orgasm, satisfaction, and pain during intercourse) (Symptoms, feelings, routine daily activities, sports activities, work and school, personal relationships, and treatment)	50	Female sexual function index (FSFI) Dermatology life quality index (DLQI)	April to October 2016	Married women with GWs that referred to the outpatient clinics of dermatology and andragogy department and gynecology and obstetrics department	None	++
Nahidi et al. [19]	Iran	18–64	Sexual desire, arousal, lubrication, orgasm, satisfaction, and pain during intercourse Marital satisfaction	74	Arizona Sexual Experience Scale (ASEX) ENRICH Marital Satisfaction Scale	2014	AGW patients in the Imam Reza hospital at Mashhad	AGW Healthy persons	++
Parkpinyo et al. [20]	Thailand	≥ 18	Sexual desire, arousal, lubrication, orgasm, satisfaction, and pain during intercourse	47	Thai version of Female Sexual Function Index (TFSFI)	May to December 2019	Women with GWs in the Siriraj Female STI clinic	GW patients only	++
Tas et al. [22]	Turkey	18–54	(Sexual desire, arousal, lubrication, orgasm, satisfaction, and pain during intercourse) depression	100	Arizona Sexual Experience Scale (ASEX) and BDI scale	September 2012 to April 2014	Refugees of both sexes who attended dermatology clinic	GW patients only	++
Campaneret al. [25]	Brazil	NR	Desire and sexual interest, foreplay, excitation of the woman, and harmonious interaction with partner comfort in sexual intercourse, orgasm, and sexual satisfaction	75	The Sexual Quotient-Female Version (SQ-F) Questionnaire	January 2011 to January 2012	Women referred to the Clinic of Lower Genital Tract Diseases and Colposcopy	(1) GW (2) CIN2,3	++
Dominiak-Felden et al. [27]	UK	18–64	(Worries and concerns; emotional impact; sexual impact; self-image; partner issues and transmission; interactions with doctors; and health control and life impact) (Pleasure, desire/frequency, desire/interest, Arousal/excitement, and orgasm) (Dimensions—mobility, self-care, usual activities, pain/discomfort and anxiety/depression)	842	The HPV Impact Profile [HIP] questionnaire The Cuestionario Especifico en Condilomas Acuminados [CECA] The European Quality of Life Index Version 5D [EQ-5D] the Change in Sexual Functioning Questionnaire [CSFQ] VAS	May 2008 to March 2009	Women with CIN1,2,3, VIN2,3 and GW who were referred to secondary care colposcopy and gynecology clinics	(1) Normal cervical cytology (2) Borderline nuclear abnormalities and/or mild dyskaryosis (3) CIN1/2/3 (4) VIN2/3 (5) GW (6) History of GW (7) Women in UK general population	++
	Canada	≥ 18	Health state, mobility, self-care, usual activities, pain/discomfort, and anxiety/depression, mental health, The anogenital wart (AGW)-specific psychosocial burden (Self-image Sexual impact, Partner/transmission, Worries/Concerns, Emotional Impact, Interaction doctors, Control/Life impact	272	EuroQol EQ-5D VAS (SF)-12 SF-36 short version of the Spielberg State- rate anxiety Inventory HIP	September 2006 to February 2008	Patients with a first or recurrent episode of AGW were recruited from the clinical practices of 42 physicians	(1) AGW Patients at recruitment time, 2 and 6 months later (2) age-matched Canadian norms	++
Escalaset al. [9]	Spain	20–45	Cognitive behavioral aspect, emotional experiences, psychic-physical well-being, and psychosexual sphere	454	CBA 2.0 (Cognitive Behavioral Assessment)	February 2006 to March 2007	Women with HPV	(1) HPV+ (2) HPV−	+
Kazeminejad et al. [21]	Iran	≥ 16	Physical function, social function, physical role playing, playing emotional role, mental health, vitality, physical pain, and general health Sexual impact	65	World Health Organization Quality of Life Brief Version (WHOQOL-BREF)	April 2017 to May 2018	AGW Patients referred to Boali-sina Hospital in Sari, Iran	(1) AGW patients (2) Healthy persons	+
Lee et al. [32]	South Korea	20–60	General health, sexual activity, cervical cancer screening behavior, psychosocial impact	400	HIP EQ-5D CECA VAS	July 2011 to 30 November 2011	AGW patients referred to cervical cytology screenings clinics	(1) HPV related disease (AGW, abnormal pap. CIN…) (2) Healthy persons	++
Piñeros et al. [12]	Columbia	18–45	Awareness and knowledge of HPV, sexual life, and self esteem	342	Questionnaire VAS scale	December 2009 to August 2010	AGW patients	None	+
Qi et al. [15]	China	18–65	Self-image, sexual impact, partner/transmission, worries/concerns, emotional impact, interaction with doctors, control/life impact	521	Questionnaire HIP	February to May 2008	GW patients	None	++
Woodhall et al. [28]	UK	≥ 18	impact of GWs on emotional and sexual wellbeing, economic evaluations, general health	81	Questionnaire EQ-5D EQ VAS CECA10	NR	GW patients attending the York GUM clinic during a 3-month period	(1) GW (2) UK general Population	+
Wang et al. [11]	Taiwan	18–65	Self-image, sexual impact, partner/transmission, worries/concerns, emotional impact, interaction with doctors, control/life impact	249	Questionnaire HIP	February to August 2006	Women with an HPV-related diagnosis or intervention within the past 3 months	(1) Normal pap smear (2) CIN1,2,3 (3)ASCUS, LSIL, HSIL (4) GW (5) Abnormal pap+ hr HPV+	++
Sénécal et al. [29]	Canada	≥ 18	Mobility, usual activities, self-care, pain or discomfort and anxiety or depression self-image, sexual impact, partner/transmission, worries/concerns, emotional impact, interaction with doctors, control/life impact	270	EQ-5D HIP EQ-VAS	September 2006 to February 2008	GW patients in initial or recurrent episode	(1) GW Patients at recruitment time, 2 and 6 months later (2) general Canadian, US and Alberta population	++
Vriend et al. [26]	Netherlands		Mobility, usual activities, self-care, pain or discomfort, anxiety or depression Health status Sexual activity, and emotional impacts	104	EQ-5D EQ-VAS CECA-10	February to August 2012	GW patients Referred to 9 STI clinics	(1) GW Patients (2) General population (2000–2002)	+

++Good quality

+Average quality

− Poor quality

**Table 2:** Characteristics of qualitative studies measuring sexual dysfunction and psychosexual outcomes included in the review

Reference	Country	Age (y)	Number of participants	Study design	Study population	Quality assessment score+
Jeng et al. [24]	Taiwan	20–60	20	Semi-structured interviews	Women with high-risk HPV	+
Lin et al. [31]	Taiwan	20–60	20	Semi-structured interviews	Women with high-risk HPV	+
(Mixed method study)	US	≥ 18	Qualitative phase: 52 Quantitative phase: 154	In‐depth interviews Literature review Expert panel Quantitative survey	Women HPV+ were recruited from local clinics	++

++ Good quality

+ Average quality

− Poor quality

#### Data collection process

Papers (ID Number, Date of form completion, Authors, Title, Journal, Year, Volume, Issue, Pages), Participants (number of participants, age, GW occurrence, the size of GW, duration of disease, marital status, other sociodemographic characteristics), Methods (study design, aim of study, recruitment method, recruitment setting, outcomes measured, method(s) of analysis), and Results (sexual dysfunction and its relationship with GWs; physical, emotional, psychological, and social effects of GWs; and overall psychosexual impact of GWs) (Additional file 3: Data Extraction Form).

### Data synthesis (narrative synthesis)

Differences in the study designs, and the study populations made meta-analysis impossible, so narrative synthesis was chosen as the best method to describe, compare and combine the study findings. We used Popay et al.'s framework [23].

## Results

### Study selection

**Fig. 1:**
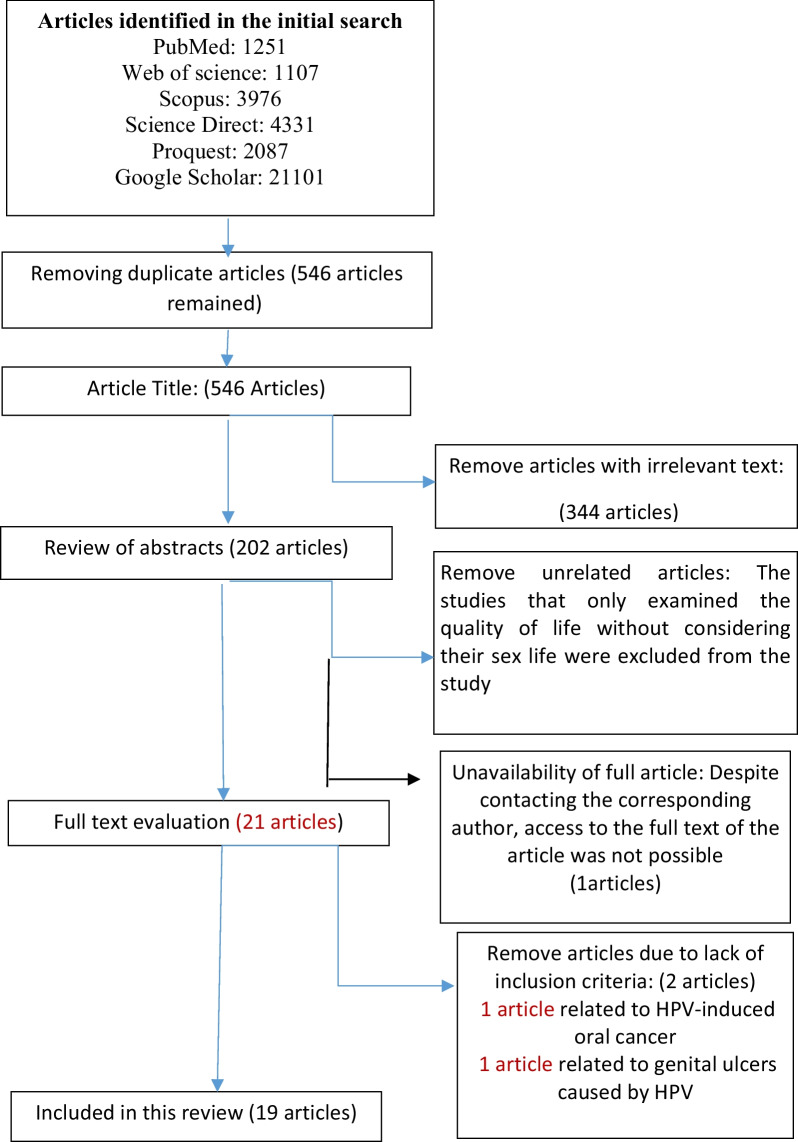
Article selection diagram

In the initial search, 33,746 articles were found. After removing duplicates and unrelated studies, 546 articles were reviewed. After reviewing the titles of the articles, 344 unrelated articles were excluded and the abstract of the remaining 202 articles was reviewed. Out of 202 studies, 181 studies were excluded for the following reasons: lack of eligibility (n = 180) (i.e. Shi, Ju-Fang, et al. "Impact of genital warts on health related quality of life in men and women in mainland China: a multicenter hospital-based cross-sectional study."BMC Public Health 12.1 (2012): 1–9) and unavailability of full text (n = 1). After evaluating the full text of the remaining 21 articles, 2 articles were excluded due to lack of inclusion criteria (1 article was related to HPV-induced oral cancer, and 1 article was related to genital ulcers caused by HPV). Finally, 19 articles were analyzed (Fig. 1).

### Study characteristics

5 articles evaluated sexual function [16, 20, 22, 24], 8 articles evaluated the psychosexual effects of GWs and HPV [11, 12, 15, 25, 26], and the remaining 6 articles evaluated the patients' quality of life, these articles also assessed the sexual effects of GWs as part of the quality of life [9, 10, 21, 27–29]. 16 articles used quantitative methods (Table 1), 2 used qualitative methods (Table 2), and 1 used mixed method (Table 2). All qualitative studies were conducted through individual interviews [24, 30, 31].

Validated scales used consisted of: Female Sexual Function Index (FSFI) (n = 2), Arizona Sexual Experience Scale (ASEX) (n = 2), The Sexual Quotient-Female Version (SQ-F) (n = 1), ENRICH Marital Satisfaction Scale (n = 1), The HPV Impact Profile (HIP) (n = 6), The Change in Sexual Functioning Questionnaire (CSFQ) (n = 1), The European Quality of Life Index Version 5D (EQ-5D) (n = 5), SF-36 (n = 1), CECA (n = 4), Cognitive Behavioral Assessment (CBA 2.0) (n = 1), and EQ-VAS (n = 5), and World Health Organization Quality of Life- Brief Version (WHOQOL-BREF) (n = 1). Based on the Quality Appraisal Checklist, most quantitative studies were well designed and had well internal (n = 9) and external validity (n = 9) (Table 1). But both qualitative studies were poorly designed (n = 2) (Table 2).

## Results of individual studies

### Prevalence of sexual dysfunction in women with GWS and HPV

Most studies confirmed that GWs, directly and/or indirectly cause sexual dysfunction in patients. 5 studies assessed the prevalence of sexual dysfunction in patients with GW [19–22, 25]. Some studies reported that 62% of women with Anal genital wart)AGW( complained of sexual dysfunction [16], whereas 72.4% complained of decreased sexual desire, 82.8% of reduced sexual intercourse, 41.4% of reduced orgasm, 57.1% of decreased sexual pleasure [25], and 70% of AGW patients suffered from decreased quality of sexual intercourse [21]. These complaints were inversely related to the level of education and income of women. Also, size of the warts, number of treatments, and the duration of treatment were inversely related to sexual dysfunction [16]. On the other hand, a study conducted on 100 refugees in Turkey reported that GWs could not change the prevalence of sexual dysfunction in refugees [22].

2 studies assessed sexual dysfunction in patients with HPV**.** The first study reported 72% decrease in libido (loss of libido), and 68% of reduced orgasms. In addition, 47% of patients had decreased libido during the year of the study. 42% of patients had less sexual intercourse, 19% were rejected by their sexual partner, and 71% reported not being able to take a new partner [9]. A Taiwanese qualitative study of 20 women with high risk HPV, found that half of the patients had problems in their sexual life, the most important of which was a decrease in the sexual desire and frequency of intercourse [24]. According to the findings, HPV and GWs can both cause sexual dysfunction in women. However, many studies confirmed that the negative impact of GWs on sexual life was more severe than that of other diseases related to HPV.

### Types of sexual dysfunction in women with GWs and associated factors

6 studies assessed types of sexual dysfunction in patients with GWs. Most studies confirmed that women with GWs experienced sexual dysfunction in all dimensions.

Some studies showed that women's sexual function decreased in the aspects of sexual desire, arousal, orgasm, sexual pain, lubrication, and sexual satisfaction. Decreased libido was more common and prominent [16, 19, 20, 25]. Women's age, duration of marriage, disease duration, HPV knowledge, number of warts, and recurrence of warts were directly related to sexual dysfunction; while there were non-significant relations between education level, place of residence, occupation, spouse infection with GW, condom use, and type of treatment, with female sexual dysfunction [19, 20]. There were also no associations between: age, multiple marriages, and marital satisfaction with sexual dysfunction [19]. Contrary to the results of previous studies, in a cross-sectional study conducted on 100 refugees with GWs in Turkey, it was observed that GWs had no effects on patients' sexual function [22]. Another study compared the sexual function of women with normal cervical cytology, borderline nuclear abnormalities, CIN1, 2, 3, VIN2, 3, GW, and history of GW, with women in the UK general population; they reported that the sexual function of GW group was similar to the general population, while in VIN group sexual dysfunction was more than the others. One of the reasons stated by the authors for this finding, was the age difference between the groups [27].

The results of a cross-sectional study suggested that the effective factors on sexual lives of men with GWs were age, and knowledge of HPV; while in women with GWs, the effective factors included education, and the location of warts. Higher education, and clitoral warts had more negative effects on women's sexual lives [12].

### Psychosexual impacts of GW and HPV

8 studies assessed the psychosexual impact of GWs**.** Some studies reported that pain, discomfort, anxiety, and depression were higher in patients with AGW. In addition, AGW had significant effects on self-image, sexual activity [10, 26, 28, 29, 32] and moderate to severe psychosexual impacts [28, 29, 32]. Women with AGW had lower scores in emotional health, and sexual activity than men with AGW [26, 32]. Women reported more severe negative effects of GWs on sexual life than men did [12, 15, 26]. In a Taiwanese study comparing the psychosexual impacts of HPV− related diseases in several groups including: normal pap smear, CIN1,2,3, ASCUS, LSIL, HSIL, GW, and abnormal pap+ hr HPV+, the results showed severe psychosexual impacts of GWs. The worst effects were on sexual life, then self-image, and worries/concerns. Generally, these psychosexual outcomes in women with GWs were four times greater than women with normal pap smears [11].

4 studies assessed psychosexual impact of HPV. Some studies reported that the first emotional reaction of women with HPV included: guilt, isolation, shame, confusion, anger, fear, worry, doubt, struggle, sadness, embarrassment, tension, fear, selfishness, helplessness, regret, disappointment, and depression [9, 30, 31], which in most women subsided over a year, while in a third of women remained for a long time [9]. In addition, some women denied the disease after being diagnosed with it, and others suspected their husbands or partners as the source of the infection. Most women asked their husbands or partners to have a checkup and use condoms. They also reduced or stopped sexual intercourse [31]. In a high-quality study, women diagnosed with HPV-related diseases, compared to those without disease had higher scores in dimensions of ‘*worries and concerns’*, *‘emotional impact’* and *‘partner’s issues and transmission’* which indicates more severe psychological impacts. Also 31% of women with HPV-related diseases reported moderate to severe anxiety and depression, which was significantly different from the normal control group [32]. About condom use in women with HPV, 39.2% reported that they did not use condoms at all, 16.9% used condoms occasionally, and only 12.8% used condoms regularly [30].

3 studies assessed worries/concerns with GWs. One of the most important concerns of patients with AGW is the fear of transmitting the disease to their sexual partners [10, 11]. An English study reported that patients with GWs suggested the highest level of agreement with the statements: *‘‘I am anxious to know whether I am going to recover from the infection for good’’ ‘‘I feel worried during sexual relations’’* and *‘‘I worry about whether the warts will get worse or whether there will be some complications’’* [28].

2 studies assessed worries/concerns in women with HPV. 73% of women with HPV were worried about transmitting the disease to their sexual partner (18, 32), and 57% of them were worried about the judgment of other people [9].

A Taiwanese qualitative study of 20 women with high risk HPV reported that the participants' emotional relationships were not generally affected by the infection, although their sex lives were severely affected [24].

## Discussion

This review analyzes the results of existing literature on sexual dysfunction in women with GWs. Considering that the explanation of experiences and perceptions of patients is better achieved through qualitative studies, and quantitative measures are mostly inadequate in this regard, but generally there are few studies in this field, the research team decided to assess both qualitative and quantitative studies related to sexual impacts of GWs and HPV.

The findings of this review confirmed the hypothesis that GWs directly and/or indirectly cause sexual dysfunction and have negative psychosexual impacts in affected women. The present study also showed that women with GWs experience sexual dysfunction in all dimensions. In relation to the first research question, the findings of this study showed that there is a relatively high prevalence of sexual dysfunction in women with GWs. Regarding the second research question, the findings showed that women with GWs experience sexual dysfunction in all dimensions. The results of the studies were contradictory, and our findings are both consistent and different from previous studies. Some studies showed that GWs could cause sexual dysfunction in patients [16, 19–21]. The disorder was confirmed in all domains of sexual function, including libido, arousal, orgasm, pain, and sexual satisfaction. These changes were more severe in decreased sexual desire, and number of sexual intercourse [19, 20, 25]. In contrast, the results of Parkpinyo et al. study showed that GWs had significant effects on reducing arousal, orgasm, pain, and sexual satisfaction in women, while there was no significant reduction in libido [16]. The absence of a control group in this study and not comparing GWs patients with normal population can be one of the reasons for this different finding. The Tas et al. study, also confirmed GWs had no effect on the sexual function of refugees in Turkey [22], which may be due to the specific characteristics of the study population in this research (refugees), and the lack of knowledge of refugees about the disease, their difficult living conditions, and less significance of GWs for them.

The change in the frequency of sexual intercourse is one of the most obvious changes in the sexual behavior of patients. Sometimes, this decrease in the frequency of sexual intercourse occurred in the early stages of the disease, which improved over time. Lin et al. [31] reported that many participants reduced or eliminated the number of sexual intercourses after being diagnosed with HPV. A cross-sectional study by Escalas et al. [9] showed that 72% of patients reduced their sexual intercourse after being infected with HPV, and 71% stated that HPV diagnosis had a negative effect on establishing a relationship with a new sexual partner. Jeng et al.'s [24] study confirmed that traditional Chinese women after being infected with HPV, while trying to maintain their marital relationship, wanted to ensure that the disease did not affect their role as spouses. HPV infection caused a decrease in sexual desire and the number of sexual intercourse in these women, and finally had a negative effect on the quality of the couple's sexual relations [24]. Taberna et al. [33] also reported that only 38% of HPV patients expressed that their and their partner's sexual life were not affected by HPV, and most patients had less sexual intercourse after diagnosis.

Pain during intercourse is another dimension of sexual function that few patients experience and report. It seems that this pain has a psychological aspect because it is experienced by patients who, after the diagnosis of the disease especially in the first days, have a lot of stress and restlessness, while none of the patients complain of the lesions' pain, and they had not experienced this pain before the diagnosis of the disease [17]. El-esawy et al. [20], Nahidi et al. [19] and Parkpinyo et al. [16] reported that GWs can cause pain during sexual intercourse.

In comparison to other HPV related diseases, GWs have more negative effects on the sexual life of the patients, and only one study reported that the negative impacts of VIN2, 3 on sexual function were greater than GWs. One of the reasons for this finding may be the difference in the average age of the affected women in the 2 groups mentioned [27].

Regarding the psychosexual impacts of GWs, our findings showed that GWs cause many negative psychosexual impacts in women. Almost all studies have confirmed that patients with GWs experience more severe psychosocial and psychosexual impacts [10–12, 15, 26, 28, 29, 32, 34, 35] compared to general population. Women and men with GWs reported moderate to severe negative psychosocial and psychosexual impacts [32], but GWs' negative impacts are more common in women than in men [12, 15, 26]. This finding may be due to the different personalities of men and women, as women are more emotional, they show more reactions to occurring problems, and have a more negative body image because of the unpleasant appearance of GWs. It also seems that women search more information about the disease than men, and care more about their health. Therefore, this finding is to be expected.

The appearance of warts is unpleasant, and this appearance causes discomfort in patients, especially in women. Worrying about the appearance of genital warts in patients can reduce their mental body image. Following the change in the patient's mental image of her body, the patient's self-confidence during sex decreases due to the fear of the lesions being observed by her husband or sexual partner. Therefore, it indirectly affects sexual function negatively [17]. Women suffer more from negative self-image than men do, and as a result, GWs have greater negative impacts on their sexual life. Pineros et al. [12] reported that the self-esteem of 90% of women and 62% of men decreased after the diagnosis of genital warts. In addition, 77% of women and 46% of men mentioned the negative effects of genital warts on their sexual lives [12]. Qi et al. [15] reported that women with genital warts are more vulnerable in the two dimensions of mental body image, and sexual impacts. While men with genital warts are more vulnerable in the two dimensions of sexual impacts, and interaction with the doctor [15]. Drolet et al. [10] also reported that genital warts have the most negative effect on the mental image of the body, sexual activity, and fear of transmitting the disease to the sexual partner.

In general, the most important psychosocial and psychosexual impacts in GWs affected women include negative sexual impacts, negative body image, worries, pain, discomfort, anxiety, depression, and limitation of social activities [10, 11, 26, 28, 29, 36].

Studies related to sexual dysfunction in HPV patients also reported that many HPV patients suffered from sexual dysfunction, which was more evident in the sexual desire, and intercourse frequency domains [9, 24, 31]. In addition, the results of qualitative studies that deeply explored the experiences and perceptions of HPV affected women, confirmed that patients experienced emotions including fear, anxiety, worry, anger, confusion, fear, worry, doubt, struggle, sadness, embarrassment, tension, regret, disappointment, and depression, which in most women decreased over a year, while in a third of women remained for a long time [9, 30, 31, 37].

After HPV diagnosis, some women denied it or reluctantly accepted it. A few women reported sexual routes as a source of infection, many of them suspected their husband or sexual partner as the source of infection. In some societies where men and women do not experience sex before marriage, contracting a sexually transmitted disease such as HPV or genital warts in one of the couples, can raise the suspicion of sexual infidelity. Despite the cultural differences in different societies, and the existence of different views among women about sexual infidelity, this issue is still important for women, and creates negative feelings in them. So that some patients consider their spouses' sexual infidelity as the main reason for their infection. Some patients only suspect that their spouse has committed sexual infidelity, and are not sure about it, but this doubt also disturbs the mental and psychological peace of the patients, and finally negatively affects their sexual function. In this regard, Lin et al. [31] and Mortensen et al. [38] confirmed that the suspicion of infidelity has always been one of the main concerns of women with genital warts and HPV. In addition, the fear of the husband's suspicion of the patient's infidelity was another concern expressed by the patients [31, 38]. In most quantitative studies, this aspect of psychosexual effects has been neglected, and only qualitative studies have addressed this issue.

The effects of demographic, cultural and social characteristics of women with GWs on their sexual function were also reported to be contradictory in several studies. The most important effective factors on sexual function included: age, duration of marriage, multiple marriages, level of education, place of residence, occupation, duration of illness, knowledge of HPV, number of warts, location and recurrence of warts, husband infection with GW, condom use, and type of treatment [12, 19, 20, 26, 34]. It seems that the cultural and social differences among the participants are the most important reasons for the different observed effective factors on sexual function in women with GWs.

Few studies assessed the concerns of women with GWs. The results of these studies showed that the most important concerns included fear of transmitting the disease to a sexual partner or husband, worsening of lesions, or recurrence of lesions through sexual intercourse [10, 11, 28]. In addition, the most important concerns of HPV infected women were fear of transmitting the disease, and fear of being judged by others [9, 32].

The fear of transmitting the disease to the husband or sexual partner causes anxiety. This stress reduces sexual desire and thus directly leads to sexual dysfunction [39]. Anxiety and worry about the spread or recurrence of lesions were reported in many studies, the severity of which was directly related to the number of lesions, rate of recurrence, and women's awareness of the disease. Women who had more lesions, or experienced frequent lesions reported more anxiety and worry. Women who had more information about the disease also experienced more anxiety. Another concern of women was the fear of transmitting the disease to their husbands. This concern was especially more profound among women whose husbands had no lesions, because they believed that their husbands were not infected, and they could transmit the disease to their husbands during sex [17]. The findings of some studies are also consistent with this finding [10, 11, 34, 39].

Faced with this fear and worry of disease transmission, many patients tried to have protected sex with a condom [31]. While the results of Daley et al.'s [30] study contradict this finding, Daley reported that 39.2% of HPV patients rarely use condoms. 16.9% of patients reported occasional use of condoms and only 12.8% of them used condoms regularly. However, most participants considered condoms to be an appropriate method of preventing disease transmission [30]. The authors found no studies investigating the rate of condom use among GWs affected women, or their attitudes in this regard. Therefore, it is not possible to comment on the role of condom use in the sexual intercourse of these patients.

Regarding the effects of genital warts on men's sexual function, different findings have been reported. Adeli et al. [17] reported that unlike the high percentage of women with GWs who suffered from sexual dysfunction, their husbands' sexual function was not affected in most cases. Few men reported lower sexual satisfaction due to a change in the type of intercourse (elimination or reduction of vaginal, oral, or anal intercourse) [17], while Chew et al. [40] reported an association between sexual dysfunction in men partnered with women with FSD, especially in the domains of erectile and ejaculatory function. Differences in the study population, and socio-cultural backgrounds may be the reasons for these observed differences in the findings, in the way that Adeli et al. evaluated the husbands of women with GWs, while Chew et al. evaluated the sexual partners of women with sexual dysfunction.

According to the objectives of the present study, the studies addressing cervical cancer, cervical intraepithelial neoplasia, men, and non-heterosexuals were excluded. This issue was a limitation of the present study. Therefore, it is suggested that these cases be examined in future studies.

## Conclusion

This study provides up-to-date evidence of the changes in sexual function in women with genital warts. Although the results of most studies showed that women with genital warts experienced sexual dysfunction in almost all dimensions, differences in study design and study population made it difficult to determine the specific type of disorder such as libido, or arousal disorders in these women. Based on the findings of this review, more research in this field is recommended for the future.

## Supplementary Information

**Additional file 1**. Prisma checklist.

**Additional file 2**. Search strategy.

**Additional file 3**. Data Extraction Form.

## Data Availability

Not applicable.

## Abbreviations

PRISMA-P: Preferred reporting items for systematic reviews and meta-analyses protocols
PROSPERO: International prospective register of systematic reviews
GW: Genital wart
HPV: Human papilloma virus
CIN: Cervical intraepithelial neoplasia
VIN: Valval intraepithelial neoplasia
ASCUS: Atypical squamous cells of undetermined significance
LSIL: Low grade squamous intraepithelial lesion
HSIL: High grade squamous intraepithelial lesion
FSFI: Female sexual function index
ASEX: Arizona sexual experience scale
SQ-F: The Sexual quotient-female version
HIP: The HPV impact profile
CSFQ: The change in sexual functioning questionnaire
EQ-5D: The European quality of life index version 5D
CBA 2.0: Cognitive behavioral assessment
WHOQOL-BREF: World Health Organization quality of life brief version
N/A: Not applicable
NICE: National Institute for Health and Care Excellence
