# Supplementary material for: Sexual dysfunction in women with genital warts: a systematic review
Source: BMC Womens Health. 2022 Dec 12;22:516. doi: 10.1186/s12905-022-02073-6 (PMC9743756; doi:10.1186/s12905-022-02073-6)
Supplement: Supplementary file 2 — Additional file 2. Search strategy. [file 12905_2022_2073_MOESM2_ESM.docx]

**File1: The search strategy for identifying papers**

| Genital wart | Condyloma acuminate | HPV | psychosexual outcome | Sexual Dysfunctions |
| --- | --- | --- | --- | --- |
| 1. "Genital Warts" | 1. "Condyloma acuminata" | 1. HPV 2. "Human Papilloma Virus" 3. "Human Papillomavirus" 4. Papillomavirus Infections | 1. Psychosexual impact 2. Psychosexual outcome 3. Psychosocial impact 4. Psychosocial outcome 5. Psych 6. Psychosexual Disorders 7. Quality of Life" | 1. Female Sexual Dysfunctions 2. Sex Impact 3. Sex Function 4. sexual desire 5. arousal 6. Lubrication 7. Orgasm 8. satisfaction 9. pain during intercourse |
| **SEARCH COMBINATIONS** | **SEARCH COMBINATIONS** | **SEARCH COMBINATIONS** | **SEARCH COMBINATIONS** | **SEARCH COMBINATIONS** |
| **1 or 2**  **1 AND 7**  **1 AND 8**  **1 AND 9**  **1 AND 10**  **1 AND 11**  **1 AND 12**  **1 AND 13**  **1 AND 14** | 1 or 2  2 AND 7  2 AND 8  2 AND 9  2 AND 10 | 1 or 2 or 3 or 4 or 5 or 6  3 AND 7  3 AND 8  3 AND 9  3 AND 10  4 AND 7  4 AND 8  4 AND 9  4 AND 10  5 AND …  …… | 7 or 8 or 9 or 10 or 11or 12 or 13  1 AND 7  1 AND 8  1 AND 9  1 AND 10  1 AND 11  1 AND 12  1 AND 13  2 AND 7  …….. | 14 or 15or 16 or 17 or 18 or 19 or 20 or 21 or 22  1 AND 14  1 AND 15  1 AND 16  1 AND 17  1 AND 18  1 AND 19  1 AND 20  1 AND 21  1 AND 22  2 AND 14  ……… |
