# Supplementary material for: Sexual dysfunction in women with genital warts: a systematic review
Source: BMC Womens Health. 2022 Dec 12;22:516. doi: 10.1186/s12905-022-02073-6 (PMC9743756; doi:10.1186/s12905-022-02073-6)
Supplement: Supplementary file 3 — Additional file 3. Data Extraction Form. [file 12905_2022_2073_MOESM3_ESM.docx]

Supporting Information
Data Extraction Form

Sexual dysfunction in women with genital warts: A systematic review

| ID Number (on Excel spreadsheet) |  |
| --- | --- |
| Date form completed |  |
| Authors |  |
| Title |  |
| Journal |  |
| Year |  |
| Volume |  |
| Issue |  |
| Pages |  |
| **Participants** |  |
| Genital wart status determined? | YES NO |
| the extent of genital wart |  |
| Duration of infection |  |
| marital status |  |
| Number of participants |  |
| Age range of participants |  |
| Gender of participants |  |
| Other relevant sociodemographic |  |
| **Methods** |  |
| Study design |  |
| Aim of study |  |
| Recruitment method |  |
| Recruitment setting |  |
| Outcomes measured |  |
| Method(s) of analysis |  |
| **Results** |  |
| Sexual dysfunction reported. | YES NO |
| If yes, give summary of results |  |
| relationship and frequency of sexual dysfunction with genital warts | YES NO |
| If yes, give summary of results |  |
| Psychosexual impact of genital wart | YES NO |
| If yes, give summary of results |  |
| physical, emotional, psychological and social impacts of genital warts | YES NO |
| If yes, give summary of results |  |
| Other notes |  |
